# Associations between perceived physical literacy and physical fitness tests among the adult population

**DOI:** 10.3389/fspor.2026.1783118

**Published:** 2026-03-09

**Authors:** Peter Holler, Sandra Dohr, Silvia Tuttner, Frank Michael Amort, Mireille van Poppel, Johannes Jaunig

**Affiliations:** 1 Department of Human Movement Science, Sport and Health, University of Graz, Graz, Austria; 2 FH JOANNEUM—University of Applied Sciences, Institute of Health and Tourism Management, Bad Gleichenberg, Austria; 3 Sport Science Laboratory, FH JOANNEUM—University of Applied Sciences, Bad Gleichenberg, Austria; 4 Geriatric Health Centres of the City of Graz, Albert Schweitzer Institute for Geriatrics and Gerontology, Graz, Austria; 5 Department of Management, University of Klagenfurt, Klagenfurt, Austria

**Keywords:** adults, cross-sectional study, physical competence, physical fitness, physical literacy, perceived physical literacy, physical activity, fitness tests

## Abstract

**Introduction:**

The multidimensional concept of physical literacy (PL) is increasingly recognized as a holistic framework supporting lifelong physical activity. Although research is growing, empirical evidence remains limited and focused mainly on children and adolescents. In adults the relationship between subjectively measured (perceived) PL and objectively measured physical fitness tests is still unclear. The aim of this study was therefore to examine associations between overall and domain-specific perceived PL and multiple components of physical fitness in an adult population.

**Methods:**

An exploratory cross-sectional study was conducted with 180 adults (60% women; mean age 40.71 ± 15.79 years), recruited during community-based physical activity education events in rural Austria. Perceived PL was assessed using the Perceived Physical Literacy Questionnaire (PPLQ), which captures six domains: motivation, confidence, physical competence, knowledge, understanding, and physical activity behavior. Physical fitness was evaluated through a series of field-based tests assessing cardiorespiratory fitness, muscular strength, flexibility of the upper and lower extremities, balance, and reaction time. Spearman correlation coefficients were calculated for the total sample and subsamples stratified by gender and age (<45/ ≥ 45 years). In line with the exploratory nature of the study, interpretation focused exclusively on effect sizes following Cohen's conventions.

**Results:**

Across all fitness parameters, the strongest correlations emerged for overall PL and the physical competence domain, followed by the physical activity behavior and confidence domains. In turn, the weakest correlations were observed for the motivation, knowledge, and understanding domains. Overall, correlations ranged predominantly from small-to-moderate, with notable variation across gender- and age-specific subgroups; in men and younger adults, correlations were generally stronger.

**Conclusion:**

This study provides new insights into the relationship between overall/domain-specific perceived PL and physical fitness tests in the adult population, particularly regarding less commonly explored fitness parameters such as upper extremity flexibility, reaction time, and balance. Given the exploratory nature of the study, future research with larger, more diverse, and probability-based samples is needed to strengthen both the internal and external validity of these findings.

## 1 Introduction

There is a substantial body of evidence highlighting beneficial physiological (1, 2) and psychological (3, 4) effects of physical activity (PA). Yet, about a third of adults globally do not engage sufficiently in PA, with even higher inactivity rates in Western countries (5, 6) increasing their risk of adverse health outcomes (1). To combat this, the World Health Organization introduced the “Global Action Plan on Physical Activity 2018–2030”, targeting a 15% reduction in global physical inactivity by 2030 (7). Central to this plan is the concept of physical literacy (PL) —a concept that has received increasing attention over the last two decades (8, 9) as it is considered a pathway for lifelong PA participation (10, 11). Originated by Margaret Whitehead (12), and later defined by the International Physical Literacy Association (IPLA), PL is the “motivation, confidence, physical competence, knowledge and understanding to value and take responsibility for engagement in physical activities for life” (13). These domains are not regarded as separate entities but as mutually reinforcing and intertwined (14–16), positioning PL as a mind-body integrated, holistic framework for PA (9, 17). PL is further framed as a “cradle to grave” concept (9), with individuals experiencing unique PL journeys shaped through interaction with their environment (18). Despite this lifespan-oriented perspective, PL research has predominantly focused on children and adolescents in school settings, whereas adults have received comparatively little attention (19). From a broader health perspective, PL is theoretically linked to a wide range of health-related outcomes due to its emphasis on sustained PA (14, 20); however, empirical evidence examining these associations in adult populations remains scarce (21). More specifically, there is a notable lack of studies in adults investigating the relationship between PL and objectively assessed fitness. This gap is of substantial public health relevance, as physical fitness constitutes a well-established intermediate marker through which PL may function as a key mechanism linking sustained PA to long-term health benefits and disease prevention (21, 22). Overall, the field of PL research is still in an early and evolving stage, characterized by scarce empirical evidence and considerable heterogeneity in conceptual frameworks, measurement instruments, and interpretations of the construct (23–26).

Despite ongoing conceptual debates, there is a broad consensus that physical competence constitutes a non-negotiable core domain of PL (27, 28). Drawing inspiration on Whitehead's work (11, 29), physical competence is commonly described as an individual's ability to acquire movement skills and patterns, and the capacity to engage in movements of varying intensities and durations (e.g., 30, 31). An adequate level of physical competence enables participation in diverse activities across multiple environments (11) —thus underpins the sustained physically active lifestyle central to PL (20). Depending on the age group being addressed, approaches to operationalizing physical competence within the PL concept vary, both in content and methodology. In children and adolescents, the most frequently used indicator of physical competence is (fundamental) movement skills proficiency, often supplemented by physical fitness assessments (32). These assessments typically rely on objective tests, which are less susceptible to bias than self-reports (33), but are time-intensive and often require substantial resources, including specialized equipment, adequate space, and trained assessors (34, 35). Consequently, their use in large-scale studies is often limited to school contexts, where such assessments are embedded in physical education curricula (36) and appropriate resources are available [e.g., physical education teachers can serve as test assessors (37)]. In adults, self-reported measures of physical competence are generally considered more feasible, as they are easily integrated into surveys, require fewer resources, and ensure participant safety—particularly for individuals with underlying health conditions (33, 35). Accordingly, the few existing PL instruments for adults predominantly assess physical competence subjectively via self-report subscales within multidimensional PL questionnaires (34).

The Perceived Physical Literacy Questionnaire (PPLQ (38),) is a holistic self-report PL instrument for adults, focusing on endurance and strength-related fitness as proxies for physical competence. As a time-efficient PL questionnaire intended for large-scale use, the prioritization of these parameters reflects a necessary trade-off associated with a limited number of items per domain. This approach acknowledges that endurance and strength are not the only contributors to the competence domain, but they are most essential for adults to sustain PA engagement. Maintaining or improving maximum oxygen uptake (VO_2max_) and muscle mass—key indicators of endurance and strength—is essential in adulthood, not only for managing daily and occupational demands without excessive fatigue or injury risk, but also for engaging in exercise and sports with enjoyment and without swift exhaustion (39). This is especially relevant given adults' preference for leisure-time activities such as walking, running, and cycling, which primarily depend on these fitness attributes (40). Moreover, higher levels of endurance- and strength-related fitness are associated with a reduced risks of a chronic diseases (41, 42), which often act as barriers to PA participation (43). By jointly promoting these fitness components (44), adults may mitigate disease risk and support long-term progression along their individual PL journey.

To date, evidence on the health-related benefits of fitness in adults has been derived almost exclusively from objective laboratory- or field-based assessments (45, 46). By contrast, the physical competence domain of the PPLQ relies entirely on self-report, leaving unresolved the extent to which its outcomes are comparable to objectively measured fitness. Although a recent meta-analysis reported a moderately high mean correlation (*r* = 0.38) between self-reported and objectively assessed fitness, the pronounced heterogeneity across studies suggests substantial variability in this relationship (47). Moreover, while age and sex have been identified as potential moderators of this relationship (47, 48), the existing evidence is largely based on younger adult samples (47), thereby substantially constraining its generalizability across the adult lifespan. This gap highlights the need for further investigation, particularly with respect to the PPLQ within the broader PL framework. As PL is conceptualized as a constellation of interrelated and mutually reinforcing domains (14–16), an exclusive focus on physical competence may yield only a partial understanding of fitness as a foundation for sustaining lifelong PA. From this perspective, examining associations between overall and domain-specific PL—encompassing motivation, confidence, physical competence, knowledge, understanding, and PA behavior—and objectively measured fitness may offer a more comprehensive understanding. However, empirical research exploring links between perceived overall/domain-specific PL and objective fitness remains scarce, with existing studies restricted to children, adolescents, or college students (49–55). Hence, robust evidence from general adult populations, particularly across a broad range of fitness components, is notably lacking. Addressing this critical gap, the present study aimed to examine associations between overall/domain-specific perceived PL and objectively measured fitness tests in the general adult population. To gain a more comprehensive understanding of this relationship, we adopted an exploratory approach that moved beyond the traditional focus on endurance [i.e., cardiorespiratory fitness (CRF)], strength and flexibility (47), to also explore indicators of balance and reaction time, which are becoming increasingly relevant for aging adults (56, 57).

## 2 Materials and methods

### 2.1 Design, participants, and procedure

This cross-sectional study was embedded in the project MOVEluencer (58), which aimed to promote PA during the COVID-19 pandemic in rural Styria, a province in Austria. Data was collected between March and April 2022. Overall/domain-specific PL was assessed with the paper-pencil version of the PPLQ (38). Fitness was determined by conducting field-based tests for CRF, muscular strength, flexibility (for both upper and lower extremity), balance, and complex hand-foot reaction time. In addition, we gathered data on age, education level and self-reported weight and height [used to calculate body-mass-index (BMI, kg/m^2^)]. The inclusion criteria for participants were (i) an age between 18 and 65 years, (ii) fluent in German language and (iii) the physical ability to participate in the fitness tests, as determined by the Physical Activity Readiness Questionnaire (59). In case of any evidence of a risk factor or injuries, individuals were not approved for the study. All participants gave their written informed consent before any study-related measurements. Ethical approval was obtained from the Research Ethics Committee of the University of Graz, Styria, Austria (GZ. 39/36/63 ex 2021/22). Participants were recruited at “PA education events”, which were held as part of the project MOVEluencer in six rural communities (58). Those events featured various stations utilizing an interactive learning approach to improve the population's awareness of active mobility and were conducted on one weekend day at a crowded public place (e.g., main square or marketplace) in each community. Social media campaigns were used to promote the events. Residents who attended the events were recruited to participate in the study through personal contact. Eligible study participants first completed the fitness tests and subsequently the PPLQ. Thereby, both the fitness tests and the completion of the PPLQ were conducted outdoors in areas protected from wind and direct sunlight, such as covered pergolas or partially open party tents. For compensation, each participant received a folder with his/her individual results regarding the fitness tests and recommendations for the improvement of the evaluated fitness parameters.

### 2.2 Measurements

#### 2.2.1 Physical fitness tests

We conducted a series of six field-based tests to evaluate physical fitness. All six tests were conducted in the order in which they are described below.

##### 2.2.1.1 Cardiorespiratory fitness

The Queens College Step Test was used to estimate VO_2max_ as an indicator of CRF (60). This test is valid, and time-effective (i.e., execution time around three minutes) with submaximal exertion (61). Following a standardized procedure (60), participants were asked to step onto and down from a step platform of 41.3 cm (16.26 inches) height for three minutes at a consistent pace set by a metronome (88 and 96 beats per minute for women and men, respectively). Subsequently, after a break of five seconds, heart rate was measured via radial palpation for 15 s and multiplied by four. Based on the obtained result, VO_2max_ was estimated using the formulas recommend by McArdle et al. (60), with a higher VO_2max_ indicating a better CRF.

##### 2.2.1.2 Muscular strength

We employed the Jamar handgrip dynamometer (Model J00105; Sammons Preston, Bolingbrook, Illinois) to assess grip strength (62), a commonly used measure that has been associated with muscular strength across different settings and age groups (63–65). Following established procedures, participants sat on a chair with the shoulder joint of the tested arm adducted, their elbow flexed at 90 degrees and their forearm and wrist held in a neutral position. Subsequently, they were instructed to squeeze the dynamometer as strong as possible. The better score (measured in kg) from two attempts with the dominant hand was selected, with a one-minute break between them. A higher test score indicated better muscular strength.

##### 2.2.1.3 Flexibility (lower extremity)

We used the classic sit-and-reach-test to determine participants' flexibility of lower extremity, particularly in the hamstring muscles (66, 67). A previous meta-analysis on reliability and validity of this test reported satisfactory results (67). The test procedure involved sitting on the floor with closed legs extended straight ahead and feet positioned against a box. Participants were instructed to reach forward as far as possible while keeping their knees fully extended. After reaching the final position, the distance between the foot sole (=zero mark) and the fingertips was measured, with higher values indicating a better flexibility of the lower extremity.

##### 2.2.1.4 Flexibility (upper extremity)

The shoulder mobility test from the Functional Movement Screen (FMS) test battery was used as an indicator of upper extremity flexibility (68). The test measures the bilateral range of motion of the shoulder joint. Following the recommended procedure, participants had to stand upright and maintain a fist with both hands (thumb in fist). Then, they were instructed to place both fists on the back by assuming a maximal adducted, extended, and internally rotated position with one shoulder and a maximal abducted, flexed, and externally rotated position with the other. The distance between the two closest bony prominences was measured. In contrast to the FMS scoring system (69), we used the raw measurement values for our study (i.e., the measured values were not converted to the four basic scores), with higher values indicating a better shoulder mobility. Participants made two attempts for both opposite fist placement (i.e., four trials in total), whereby the mean of each of the best attempts were used for our analysis.

##### 2.2.1.5 Balance

We employed the MFT-S3-Check (MFT Bodyteamwork GmbH, Kirchberg, Austria) to evaluate balance (70). This reliable and valid test system consists of an unstable uniaxial platform (55 cm diameter), equipped with an integrated sensor and corresponding software. The platform is linked to a base plate via the sagittal axis and allows a lateral tilt of up to 12 degrees to both sides (referred as left-right movements). In the test, participants were instructed to stand on the platform as horizontal and controlled as possible for two 30 s intervals, separated by a 30 s break. Prior to the measurement, there was a familiarization phase of 15 s. Based on the frequency and magnitude of the left-right-oscillations, the system calculated a stability index from 0 to 10, with 10 being the best possible value.

##### 2.2.1.6 Complex hand-foot reaction time

The Match-4-Point test from the Talent Diagnostic System was applied to determine participants' complex hand-foot reaction time at visual stimulation (71). A previous study on the reliability and validity of this test reported satisfactory results (72). The corresponding instrument consists of three plates: two for the hands, positioned on a table one meter in height, and one designated for the left and right foot, placed two-thirds underneath the table. The plates are connected to a laptop equipped with dedicated software. In the test, a series of 20 random visual stimuli appeared on the laptop screen. Participants were instructed to stand in front of the screen and touch the correct plates as quickly as possible using their left or right hand or foot, depending on the stimulus provided. By averaging the reaction time to each stimulus, the system calculated an index for complex hand-foot reaction time, with lower values indicating a better test performance. For brevity, we labeled this fitness parameter only as “reaction time” in the following.

#### 2.2.2 Perceived physical literacy

We employed the PPLQ to assess perceived PL in this study, which was specifically designed for the adult population in German speaking countries (38). Conceptualized according to the prominent IPLA definition (13), the 24-item questionnaire enables a comprehensive and differentiated measurement of six PL domains (motivation, confidence, physical competence, knowledge, understanding and PA behavior), while also providing an overall PL score. Sufficient levels of construct validity and reliability for the PPLQ have been established previously (38). The domain scores ranging between 0 and 100 were calculated for each of the six domains, with higher values representing a greater domain proficiency. The overall PL score is a composite calculation in which each of the six domains is weighted equally with 16.67%. This composite score ranges also between 0 and 100, with higher values indicating a greater PL.

### 2.3 Statistical analysis

We excluded participants who had no valid data for any physical fitness parameter or had missing values for all items in one or more domains of the PPLQ. Additionally, regarding the six physical fitness tests, individual values were considered invalid and excluded in case of non-compliance with the test protocol (e.g., inconsistent stepping pace during the Queens College Step Test). These missing values were not imputed. Only remaining missing values for the PPLQ were addressed using multiple imputation, whereby the results of five imputed data sets were averaged to have one single complete data set (73). Descriptive statistics are presented using mean and standard deviation. We used spearman correlation coefficients (r_s_), with pairwise deletion in case of missing data, to determine the association between perceived PL and physical fitness parameters. Given the exploratory nature of this study, we refrained from drawing conclusions about the presence or absence of associations based on statistical significance (*p*-values), in line with prior methodological recommendations (74, 75). Instead, our analysis relied exclusively on the effect sizes of the correlation coefficients obtained, with interpretation following Cohen's (1988) conventions: 0.10 to 0.29, “small”; 0.30 to 0.49, “moderate”; and ≥ 0.50, “large” (76). The correlation analyses were conducted for the total sample as well as for subsamples stratified by gender (men/women) and age (<45 years/ ≥ 45 years). Rather than using the median for age stratification (=42.5 years), we applied the latter cut-off, which is commonly used in research for age categorization in the adulthood population (77). To ensure a consistent interpretation of the results, we reversed the polarity of the correlation coefficients for reaction time, as it was the only fitness parameter inversely poled. To compare corresponding correlation coefficients between the stratified groups, Fisher's z-transformation and statistical significance testing (*p* < 0.05, two tailed) were applied. The results from both the total sample and the subsamples were displayed together using heat maps created with GraphPad Prism version 7 (GraphPad Software, La Jolla, CA, USA). The statistical analyses were performed using SPSS Statistics version 29 (IBM Corp, Armonk, NY, USA).

## 3 Results

Of 280 individuals invited, 260 agreed to participate (response rate = 93%) and were screened for eligibility. During eligibility screening, 63 individuals were excluded prior to study enrollment because they were outside the defined age range of 18 to 65 years (*n* = 25) or had medical conditions that precluded safe participation (*n* = 38). Of the 197 participants enrolled in the study, we subsequently excluded 16 individuals as they had missing values for all items in one or more domains of the PPLQ and one other individual due to implausible data (i.e., same rating to all items of the PPLQ). As a result, the data of 180 participants were analyzed. Participant characteristics for the total sample and the stratified subsample are provided in Table 1. Notably, when comparing the corresponding subsamples, we observed only statistically significant differences for education level (*p* < 0.001) between the age-specific subsamples (<45 years and ≥45 years), with a tendency towards a higher education level among the younger participants.

**Table 1:** Participants characteristics.

Variables	Total	Female	Male	Age < 45 yrs	Age ≥ 45 yrs
*N*	180	108	72	91	89
Age (years)^a^	40.71 ± 15.79	40.63 ± 15.98	40.83 ± 15.62	26.36 ± 6.74	55.38 ± 5.51
BMI (kg/m^2^)^b^	24.32 ± 3.93	23.34 ± 3,37	25.78 ± 3.39	23.37 ± 3.57	25.28 ± 4.06
Education level (%)^c^^,^^#^					
Compulsory school	8 (5%)	5 (5%)	3 (4%)	3 (3%)	5 (6%)
Apprenticeship	38 (21%)	20 (19%)	18 (25%)	9 (10%)	29 (33%)
Vocational middle school	25 (14%)	18 (17%)	7 (10%)	5 (6%)	20 (23%)
High school (A-Level)	56 (31%)	32 (30%)	24 (33%)	42 (47%)	14 (16%)
University-related teaching institution	14 (8%)	10 (9%)	4 (6%)	3 (3%)	11 (12%)
University	37 (21%)	21 (20%)	16 (22%)	27 (30%)	10 (11%)

^a^ No missing data.

^b^ Missing data for one female participant younger than 45 years.

^c^ Missing data for two female participants, both younger than 45 years.

# Significant difference (*p* < 0.001) between younger (age < 45 yrs) and older participants (age ≥ 45 yrs).

Among the included participants, the percentage of imputed missing values for the PPLQ was 0.15% (8 values). No values were imputed for the fitness parameters, with a total of 47 missing or invalid values (4.35%). Table 2 provides the descriptive characteristics including the numbers of valid cases for all outcome variables in all subsamples. For the gender-specific subsamples, we observed that men exhibited significantly higher scores in physical competence (*p* < 0.001), whereas women scored higher in the domains of knowledge and understanding (both *p* < 0.05). Significant disparities were also noted in CRF and muscular strength, with men demonstrating higher test performance in both (*p* < 0.001). In contrast, women showed greater flexibility in both upper and lower extremities (*p* < 0.001). Within the age-specific subgroups, participants younger than 45 years had statistically significant higher scores on overall PL (*p* < 0.001) and scored higher in the domains of confidence and physical competence (both *p* < 0.001), alongside understanding and PA behavior (both *p* < 0.05). In terms of objective fitness parameters, the younger subgroup also performed better in upper and lower extremity flexibility (*p* < 0.001 and *p* < 0.01 respectively) and balance and reaction time (both *p* < 0.001).

**Table 2:** Descriptive statistics of all study variables.

Variables	Total		Female		Male		Age < 45 yrs		Age ≥ 45 yrs	
	*n*	*M* ± *SD*	*n*	*M* ± *SD*	*n*	*M* ± *SD*	*n*	*M* ± *SD*	*n*	*M* ± *SD*
Physical Literacy (all scores 0 to 100)	180	73.63 ± 14.17	108	73.68 ± 12.64	72	73.54 ± 16.28	91	77.31 ± 12.53	89	69.55 ± 14.65***
Motivation	180	84.22 ± 14.48	108	85.56 ± 12.52	72	82.22 ± 16.90	91	85.13 ± 14.89	89	83.30 ± 14.07
Confidence	180	66.00 ± 22.74	108	64.82 ± 21.75	72	67.78 ± 24.20	91	72.53 ± 20.59	89	59.33 ± 23.00***
Physical Competence	180	66.18 ± 18.41	108	61.75 ± 17.48	72	72.84 ± 17.88***	91	70.75 ± 17.41	89	61.51 ± 18.33***
Knowledge	180	64.03 ± 23.88	108	67.13 ± 23.22	72	59.38 ± 24.26*	91	65.93 ± 23.06	89	62.08 ± 24.68
Understanding	180	89.89 ± 13.62	108	92.72 ± 9.36	72	85.65 ± 17.47*	91	92.31 ± 10.65	89	87.42 ± 15.78*
Physical Activity Behavior	180	71.43 ± 37.98	108	70.13 ± 39.13	72	73.38 ± 39.41	91	79.00 ± 32.47	89	63.69 ± 41.67*
Physical Fitness										
CRF (mL/min/kg)	156	44.65 ± 8.41	93	40.42 ± 4.72	63	50.90 ± 8.80***	84	45.28 ± 7.89	72	43.92 ± 8.98
Strength (kg)	180	40.83 ± 11.07	108	33.98 ± 6.32	72	51.13 ± 8.34***	91	42.40 ± 10.87	89	39.24 ± 11.09
Lower Extremity Flexibility (cm)	174	2.28 ± 10.22	106	4.45 ± 9.46	68	−1.12 ± 10.51***	88	4.67 ± 9.80	86	−0.17 ± 10.12**
Upper Extremity Flexibility (cm)	174	−14.85 ± 8.85	106	−12.35 ± 7.31	68	−18.75 ± 9.64***	89	−11.95 ± 6.27	85	−17.89 ± 10.09***
Balance (score 0 to 10)	177	5.53 ± 1.56	108	5.69 ± 1.58	69	5.29 ± 1.50	90	6.18 ± 1.35	87	4.86 ± 1.47***
Reaction Time (sec)	172	1.22 ± 0.36	108	1.24 ± 0.35	64	1.18 ± 0.37	85	1.03 ± 0.19	87	1.40 ± 0.39***

CRF, cardiorespiratory fitness.

* significant difference (*p* < .05) between corresponding age/gender group.

** significant difference (*p* < .01) between corresponding age/gender group.

*** significant difference (*p* < 0.001) between corresponding age/gender group.

### 3.1 Perceived physical literacy and objective fitness

**Figure 1:**
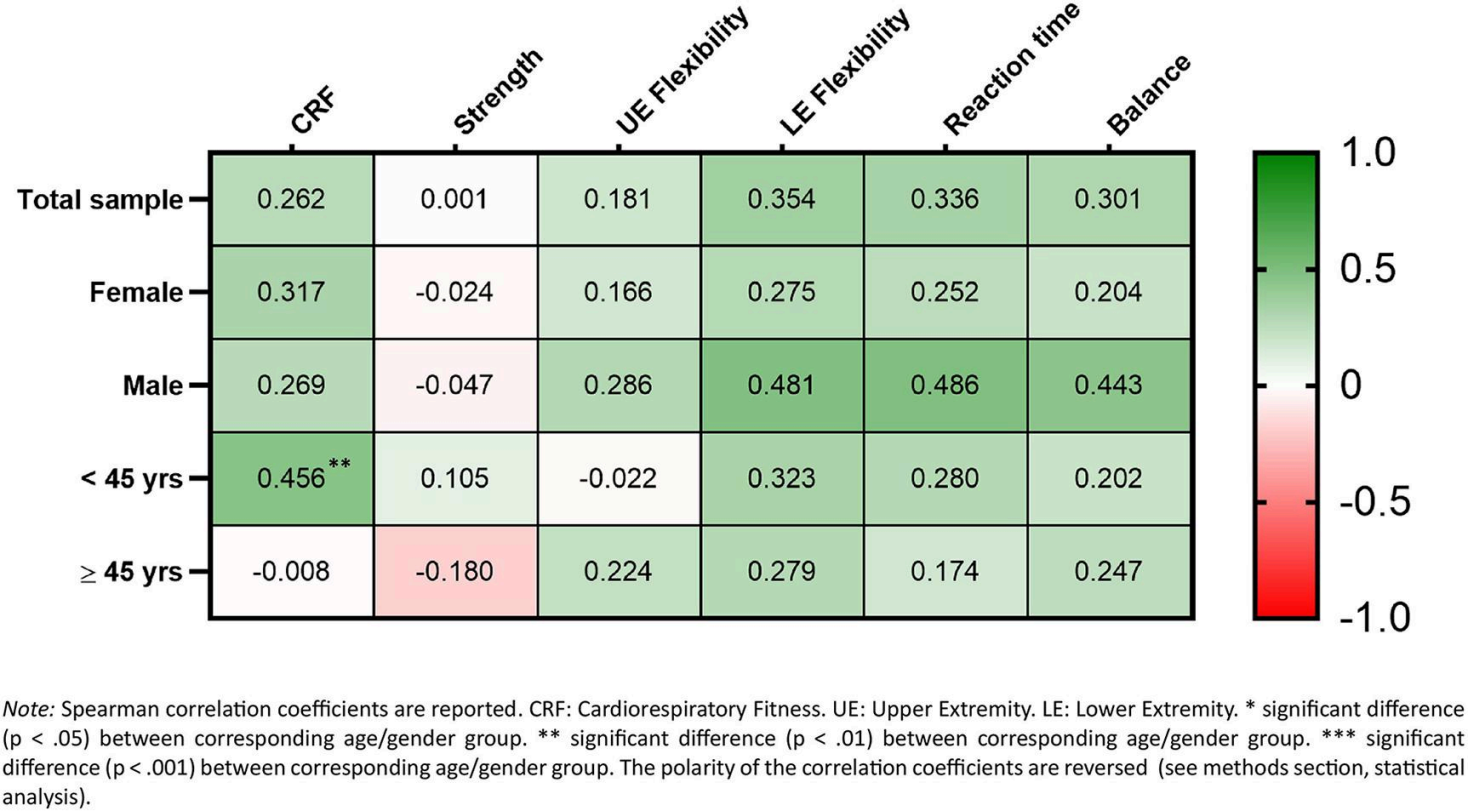
Correlation between perceived physical literacy and objective fitness.

The correlations between perceived overall PL and fitness parameters for the total sample and all subsamples are shown in Figure 1. In the total sample, small-to-moderate correlations were observed between PL and all fitness parameters except muscular strength (*r_s_* = 0.001), with coefficients ranging from *r_s_* = 0.181 to 0.354 and the strongest correlation observed for lower extremity flexibility. This pattern was consistent across gender-specific subsamples, with men showing slightly stronger correlations—particularly moderate correlations for lower extremity flexibility, reaction time, and balance (*r_s_* = 0.443 to 0.486)—compared with small correlations in women; however, none of these gender differences were statistically significant. Across age-specific subsamples, small-to-moderate correlations were evident for lower extremity flexibility, reaction time, and balance (*r_s_* = 0.174 to 0.323). A moderate correlation between PL and CRF was observed in younger but not in older participants (*r_s_* = 0.456 vs*. r_s_* = −0.008), with a significant between-group difference (*p* = 0.003). Conversely, a small correlation between PL and upper extremity flexibility was present only in older participants (*r_s_* = 0.224), while muscular strength showed a small positive correlation in younger participants (*r_s_* = 0.105) and a small inverse correlation in older participants (*r_s_* = −0.180), without significant age-group differences.

### 3.2 Domains of physical literacy and objective fitness

#### 3.2.1 Domain motivation

**Figure 2:**
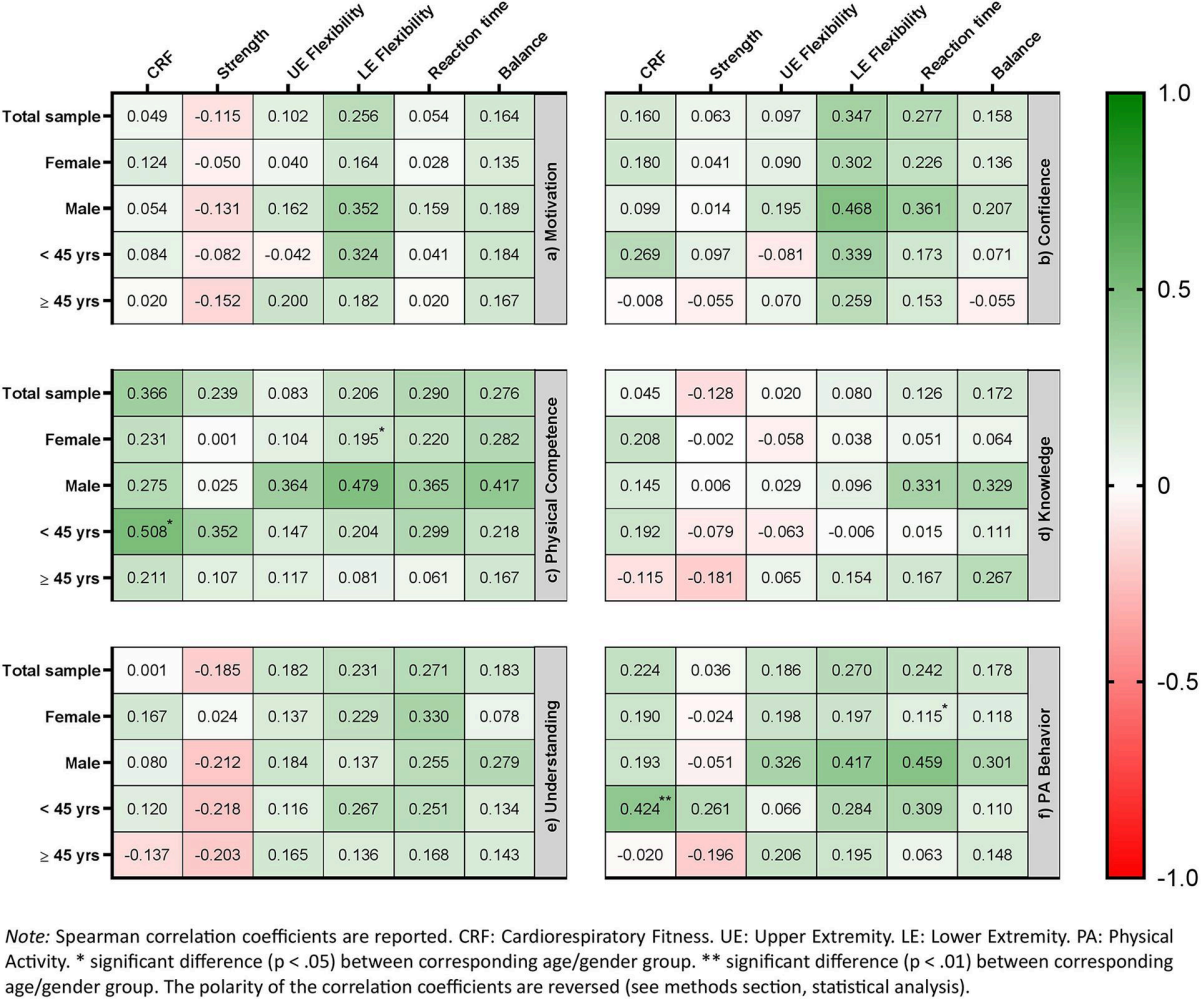
Correlation between domains of physical literacy and objective fitness.

In the total sample, small positive correlations were found between motivation and balance as well as upper and lower extremity flexibility (*r_s_* = 0.164, 0.102, and 0.256, respectively). A small inverse correlation was observed for muscular strength (*r_s_* = −0.115), whereas no apparent correlations were found for CRF or reaction time. Similar patterns were observed across gender- and age-specific subsamples, with a tendency toward stronger correlations in men; however, no statistically significant group differences were detected (see Figure 2a).

#### 3.2.2 Domain confidence

Small-to-moderate correlations were observed between confidence and most fitness parameters in the total sample (*r_s_* = 0.158–0.347), except for muscular strength and upper extremity flexibility, for which no meaningful correlations were found. Overall, men tended to exhibit slightly stronger correlations, whereas similar patterns were observed across the remaining gender- and age-specific subsamples; however, no statistically significant group differences were detected (see Figure 2b).

#### 3.2.3 Domain physical competence

Except for upper extremity flexibility (*r_s_* = 0.083), small-to-moderate correlations were observed between physical competence and all fitness parameters in the total sample (*r_s_* = 0.206 to 0.366). Across the gender- and age-specific subsamples, men and younger participants generally showed stronger correlations, with a statistically significant gender difference for lower extremity flexibility (*r_s_* = 0.479 in men vs. *r_s_* = 0.195 in women; *p* = 0.041) and a significant age difference for CRF (*r_s_* = 0.508 in younger vs. *r_s_* = 0.211 in older participants; *p* = 0.035, see Figure 2c).

#### 3.2.4 Domain knowledge

In the total sample, small positive correlations were observed between knowledge and reaction time as well as balance (*r_s_* = 0.126 to 0.172), alongside a small inverse correlation with muscular strength (*r_s_* = −0.128). No meaningful correlations were found for CRF or for upper or lower extremity flexibility. Across the subsamples, correlation patterns varied slightly and showed no consistent trend, apart from a tendency toward stronger correlations in men; however, no statistically significant group differences were detected (see Figure 2d).

#### 3.2.5 Domain understanding

In the total sample, a small inverse correlation was observed between understanding and muscular strength (*r_s_* = −0.185). Except for CRF, for which no meaningful correlation was found, all remaining fitness parameters showed small positive correlations (*r_s_* = 0.182 to 0.271). This correlation pattern was largely consistent across age- and gender-specific subsamples, with no statistically significant differences observed between any subgroups (see Figure 2e).

#### 3.2.6 Domain physical activity behavior

Except for muscular strength, for which no apparent correlation was evident, small correlations were observed between PA behavior and all fitness parameters in the total sample (*r_s_* = 0.186 to 0.270). Across gender- and age-specific subsamples, men and younger participants generally showed stronger correlations, with a statistically significant gender difference for reaction time (*r_s_* = 0.459 vs. *r_s_* = 0.115; *p* = 0.018) and a significant age difference for cardiorespiratory fitness (*r_s_* = 0.428 in younger vs. *r_s_* = −0.020 in older participants; *p* = 0.008; see Figure 2f).

## 4 Discussion

The purpose of this study was to explore the association between overall/domain-specific PL and objectively measured fitness tests in the general adult population. Our approach juxtaposed the PPLQ as a perceived PL measurement tool covering six domains [i.e., motivation, confidence, physical competence, knowledge, understanding, and PA behavior (38)], with six fitness field-based tests for CRF, muscular strength, lower/upper-extremity flexibility, balance and reaction time. Generally, the highest correlations with these fitness parameters were found for domain scores of overall PL and the physical competence, succeeded by PA behavior and confidence. The motivation, knowledge, and understanding domain scores demonstrated the weakest correlations. Predominantly, the detected associations ranged from small-to-moderate (76), and varied considerably across gender and age groups, with men and younger individuals generally showing stronger associations. Beyond these observed associations between PL and fitness outcomes, age-related differences in overall/domain-specific PL were evident, with younger adults demonstrating higher overall PL scores and higher domain-specific scores for confidence, physical competence, understanding, and PA behavior compared with the older subgroup. These differences may partly reflect the generally higher fitness levels and the higher educational level of the younger subgroup in our study, as education is associated with key PL domains such as PA behavior and confidence (78, 79); however, some degree of sampling bias may have occurred due to convenience-based recruitment, potentially resulting in self-selection bias. In particular, participation may have been more attractive to younger individuals with a higher interest in PA and exercise, which warrants cautious interpretation of these cross-sectional findings, especially with regard to the lifelong nature of PL.

In the field of PL research, relatively few studies have examined associations between overall PL and objective fitness, with most focusing on children (49, 53, 80) and adolescents (50, 52, 80), leaving a clear gap in adult populations. To date, only Zhang et al. (51) and Long et al. (54) have examined this relationship in adults, evaluating perceived overall PL in relation to multiple fitness components such as aerobic fitness (i.e., CRF), muscular strength, and lower-extremity flexibility. However, both studies were limited to university student samples, allowing comparisons only with our younger subgroup. Against this background, the magnitude of correlations observed in the present study was largely comparable to previous findings, with similarly small correlations for muscular strength and moderate, somewhat stronger correlations for CRF and lower-extremity flexibility. These comparisons should nevertheless be interpreted with caution due to methodological heterogeneity, including differences in fitness assessment protocols and PL measurement instruments. In particular, overall PL scores represent composites of multiple domains, yet their structure and theoretical grounding vary substantially across instruments (34), as illustrated by differences between the PPLQ used in our study and the simplified Chinese version of the Perceived Physical Literacy Instrument applied by Zhang et al. and Long et al. (81). Collectively, these considerations underscore the importance of emphasizing domain-specific results when interpreting and comparing PL–fitness associations across studies.

In the physical competence domain of the PPLQ, the focus lies on perceived endurance and strength-related fitness (see Introduction). The most recent meta-analysis by Germain and Hausenblas (47) examining perceived and objective fitness in adults reported an overall mean correlation of *r* = .38, with the available evidence being largely confined to CRF and, to a lesser extent, muscular strength and lower-extremity flexibility. Compared with this overall effect size, the correlations observed in our study were generally lower, even for comparable objective measures such as CRF and muscular strength. However, as most studies included in that meta-analysis were based on relatively young samples, our finding of stronger correlations in younger adults—particularly for CRF (*r_s_* = 0.51) and muscular strength (*r_s_* = 0.35)—is consistent with previous evidence and theoretical considerations (47, 48). In contrast, our gender-specific analyses diverged from earlier findings reporting either no gender differences (47) or stronger correlations in women (48, 82), a discrepancy that likely reflects methodological characteristics of our study, such as convenience-based recruitment, which may have introduced selection bias and constrained sample representativeness and external validity. From a measurement perspective, the interpretation of the observed associations should further consider the content structure of the physical competence domain of the PPLQ (38). Although the subscale has been shown to be psychometrically unidimensional, it comprises an equal number of endurance- and strength-related items (two items each). This balanced item composition may have attenuated the observed correlations with the objective fitness test results of CRF and muscular strength, as the construct does not exclusively reflect a single physical capacity. Moreover, the strength-related items of the competence domain reflect muscular strength in a general, functional manner (e.g., “I have a lot of muscle power”). In contrast, muscular strength was assessed via handgrip strength, whose reported associations with general muscular strength vary in magnitude and appear to be influenced by factors such as gender and height (63–65), potentially further attenuating the observed correlations.

Within the PA behavior domain, prior research has consistently reported robust correlations between self-reported PA and specific components of physical fitness in adults, particularly CRF (83–85) and muscular strength (83, 86), whereas findings remain mixed for lower-extremity flexibility (87–89), reaction time (90, 91), and balance (88, 92, 93). Only one study among young adults examined upper-extremity flexibility and revealed no correlation with PA (88), consistent with our findings. Overall, aside from muscular strength, the correlations observed in our study were predominantly small-to-moderate, mirroring those reported in previous research. Our data further exposed a gender pattern consistent with findings from a large-scale adult study (*n* = 1032; age 20–59 years), in which men showed slightly higher correlations between self-reported PA and objective fitness than women (83). Notably, no correlation between PA and muscular strength was observed, except in younger adults. This pattern may reflect the content focus of the PA domain of the PPLQ, which primarily emphasizes endurance-related activities, with strength-oriented behaviors being minimally represented (38). Accordingly, PA scores derived from the PPLQ may align more closely with CRF than with muscular strength. In a similar vein, heterogeneity in preferred PA and exercise modalities (e.g., endurance-focused vs. strength-focused behaviors) may partially explain the observed findings. An exploratory profile-based analysis indicated a descriptive tendency toward higher PA scores in fitness profiles characterized by higher CRF; however, the observed effect was very small (see Appendix A). Moreover, given the well-established relationship between endurance-related PA and CRF (83–85), the absence of a corresponding correlation in the older subsample was unexpected. This finding may be attributable to an attenuated adaptive fitness response to PA with advancing age (92) as well as increasing inaccuracies in self-reported PA (94).

The confidence domain of the PPLQ is conceptually grounded in Bandura's theory of self-efficacy related to PA and exercise (38). A recent systematic review from Medrano-Ureña et al. (95) on physical fitness and exercise self-efficacy in adults identified four studies examining CRF in middle-aged populations, three of which reported positive correlations. In contrast, no correlation between CRF and confidence was observed in the older subsample of our study. This discrepancy, however, should be interpreted cautiously, as the reviewed studies focused exclusively on clinical populations and examined longitudinal changes in CRF and self-efficacy, whereas our study involved healthy adults and employed a cross-sectional design. Beyond this review, studies in younger adults reported predominantly small-to-moderate correlations between self-efficacy and CRF, muscular strength, and lower-extremity flexibility (96, 97). The younger adult group in our study showed small-to-moderate correlations for CRF and lower-extremity flexibility only, characterized by slightly weaker correlations for CRF and somewhat stronger correlations for flexibility relative to previous findings. In contrast, no correlation was observed between confidence and muscular strength, potentially reflecting methodological differences in strength assessment across studies.

Within the motivation domain, previous research has examined associations between PA/exercise motivation and physical fitness in adults, primarily focusing on CRF (54, 98, 99) and to a lesser extent on muscular strength (54, 100) and lower-extremity flexibility (51, 54). Overall, prior research consistently reports small-to-moderate correlations between motivation and both CRF and muscular strength, while lower-extremity flexibility appears unrelated to motivation. In contrast, our findings did not replicate these associations with CRF or muscular strength across the overall sample or stratified subgroups, except for a small correlation with CRF in women. Instead, small-to-moderate correlations were observed with lower-extremity flexibility. These discrepancies may partly reflect differences in sample characteristics, as many previous studies relied on small, homogeneous student populations, as well as methodological differences in fitness assessment and motivation measurement. In particular, whereas most comparative studies employed maximal fitness tests, the present study used the submaximal Queens College Step Test, which entails a higher standard error in estimating CRF (VO₂_max_) (101); additionally, the PPLQ motivation domain is grounded in self-determination theory (38), whereas comparative studies relied on distinct theoretical frameworks.

For the knowledge domain, most existing PL questionnaires do not assess this construct separately but subsume it under the broader domain of “knowledge and understanding” (25, 32, 34). The PPLQ is the first questionnaire to explicitly distinguish knowledge as a separate component, operationalizing it as declarative knowledge related to health-enhancing PA and exercise (38). In our study, correlations between knowledge and fitness indicators were generally absent or small across the total sample and most subgroups, except for moderate correlations with reaction time and balance in men. These findings are largely consistent with previous studies reporting no or small correlations between PA- or exercise-related knowledge and CRF, muscular strength, or lower-extremity flexibility (54, 102). Overall, evidence remains limited and largely restricted to young adult samples, underscoring the continued underrepresentation of the knowledge domain within the PL framework (27, 33, 34).

The understanding domain, which the PPLQ operationalizes separately from knowledge, reflects subjective, meaning-oriented cognition, namely individuals' appraisal of the value of lifelong PA and exercise for health and well-being (38). This construct closely aligns with the concept of cognitive attitude in psychological models such as the Theory of Planned Behavior (103). To date, no published studies have examined whether understanding, as operationalized in the PPLQ, or related constructs are associated with objectively measured fitness in adults, indicating a clear research gap. In the present study, only limited positive correlations with CRF and muscular strength were observed, whereas correlations with other fitness parameters were small. Notably, the understanding domain exhibited ceiling effects and low variance across the total sample and subgroups (see Table 2), which may have attenuated the observed correlations (104, 105).

### 4.1 Implications for practice and future research

Considering the comparison between the present findings and existing literature, several priorities for future research emerge. First, substantial heterogeneity remains in the conceptualization and measurement of overall/domain-specific PL, as well as in fitness assessment protocols, underscoring the need for greater methodological standardization to enable meaningful cross-study comparisons. Second, research on PL–fitness associations—both within and beyond the PL framework—has largely focused on young adult samples, particularly university students, leaving middle-aged adults underrepresented. Extending research into these groups and incorporating less frequently assessed fitness components, such as upper-extremity flexibility, balance, and reaction time, would help advance a more comprehensive lifespan perspective on PL. Finally, further research is warranted on the knowledge and understanding domains of PL, which remain comparatively underexplored despite being identified as gaps in earlier conceptual work (27).

Regarding the physical competence domain of the PPLQ, the conceptual distinction and overlap between perceived and objective fitness warrant further investigation. Consistent with previous research (47, 48), the correlations observed between these constructs were small-to-moderate, indicating that perceived and objective fitness are related yet represent distinct, non-interchangeable constructs. This finding has important practical implications not only for users of the PPLQ but also for PL assessment in adults more generally, as the physical competence domain—often encompassing physical fitness—is typically evaluated through self-report measures in multidimensional PL questionnaires (34). Given that evidence linking fitness to health outcomes is largely based on objectively measured fitness (45, 46), feedback provided to participants based solely on perceived fitness should therefore be interpreted with caution; for example, overestimated self-perceptions may hinder further fitness development and contribute to stagnation along an individual's PL journey (106). Where feasible, the inclusion of brief, valid, and practicable objective fitness assessments—such as those employed in the present study— may help practitioners better understand discrepancies between perceived and objective fitness and provide participants with appropriate, norm-referenced feedback. At the same time, since objective fitness testing in adult populations is often constrained by practical, logistical, and health-related considerations (33, 35), further empirical evidence is needed to clarify the relationship between perceived fitness and objectively measured health outcomes, an area in which research remains scarce. In this context, users of the PPLQ are well positioned to contribute to strengthening the evidence base linking fitness, as well as broader overall/domain-specific PL, to objective health indicators in adulthood, an area that remains both empirically limited and conceptually underdeveloped (21). Finally, discrepancies between perceived and objective fitness have frequently been attributed to psychological and social factors, including emotional states, body image, social desirability, and self-worth (106); from this perspective, holistic PL interventions may represent a particularly promising learning context, as they emphasize reflection, embodied experience, and self-awareness (107), potentially supporting individuals in developing more accurate perceptions of their own fitness levels. In this intervention context, fitness-related feedback from commercially available trackers may offer an additional practical avenue for enhancing the accuracy of fitness perceptions.

### 4.2 Limitations

Several limitations should be considered when interpreting our findings. First, the use of a convenience sample may have introduced selection bias, as individuals more interested in PA and exercise were likely overrepresented. This bias appears most evident in the understanding domain of the PPLQ, where high average scores and low variance suggest a ceiling effect. Together with small sample sizes, this limits the generalizability (external validity) of the observed correlations. Accordingly, future studies should rely on larger, more diverse, probability-based samples to address this limitation. Second, the cross-sectional design precludes causal inferences regarding the direction of associations between overall/domain-specific PL and objective fitness. Finally, certain limitations concern the measurement instruments. Although the PPLQ is validated for adults, self-report may be affected by recall bias and social desirability. Likewise, fitness parameters were assessed using field-based tests rather than standardized laboratory measures. While practical for large-scale studies, the measurement properties are inferior to laboratory gold-standard tests and more vulnerable to external influences, as exemplified by the Queens College Step Test used to estimate CRF in this study. For instance, a cardiopulmonary exercise test, as a gold-standard test for CRF, might yield more precise insights into the conceptual distinction between perceived and objectively measured fitness.

## 5 Conclusion

This study provides novel insights into associations between perceived overall/domain-specific PL and objectively measured fitness components in a general adult population. Overall, associations were predominantly small-to-moderate and tended to be stronger in men and younger adults, with the strongest relationships observed for overall PL and physical competence, followed by PA behavior and confidence, whereas motivation, knowledge, and understanding showed weaker associations. Although these findings address an important research gap, their exploratory nature and reliance on a convenience sample warrant cautious interpretation, underscoring the need for future studies using larger, more diverse, probability-based samples. Moreover, comparisons with existing literature reveal substantial methodological heterogeneity in both PL measurement and fitness assessment, as well as a continued lack of research in middle-aged adults and for less frequently assessed fitness components, such as upper-extremity flexibility, balance, and reaction time. Finally, within the physical competence domain, future studies should further clarify the conceptual distinction and overlap between perceived and objectively measured fitness, particularly in relation to health outcomes.

## Data Availability

The raw data supporting the conclusions of this article will be made available by the authors, without undue reservation.

## Appendix A. Detailed analysis for the physical activity domain

### Aim

The aim of this analysis was to examine how different fitness profiles, in terms of cardiorespiratory fitness (CRF) and muscular strength, are associated with scores in the physical activity (PA) domain of the Perceived Physical Literacy Questionnaire (PPLQ).

### Methods

The sample was stratified according to CRF and muscular strength using median splits (median CRF = 42.17 mL·min^−1^·kg^−1^; median muscular strength = 38.00 kg). This resulted in four fitness profiles: (i) low CRF–low muscular strength, (ii) low CRF–high muscular strength, (iii) high CRF–low muscular strength, and (iv) high CRF–high muscular strength. Group differences were subsequently examined, using pairwise deletion in case of missing data. Due to pronounced right-skewed distributions across all four fitness profiles (visual inspection of histograms), the Kruskal–Wallis test was applied (two-sided, α = 0.05).

### Results

The results for the PA domain score stratified by the four fitness profiles are presented in Table A1. Although the descriptive data suggests a tendency whereby higher CRF profiles are associated with higher scores in the PA domain, group differences were not statistically significant (H = 4.09, df = 3, *p* = 0.252), with a very small effect size (ε^2^ ≈ 0.007).

**Table A1:** Physical activity domain score by fitness profile.

Fitness profiles	*n*	M ± SD	Mdn
Low CRF—Low muscular strength	56	65.23 ± 40.83	91.70
Low CRF—High muscular strength	22	62.49 ± 42.55	87.00
High CRF—Low muscular strength	22	73.82 ± 34.57	95.12
High CRF—High muscular strength	56	79.24 ± 33.02	100.00

Values range from 0 to 100, with higher values indicating higher physical activity levels. M, mean; SD, standard deviation; Mdn, median. CRF, cardiorespiratory fitness.
